# Microsecond cell triple-sorting enabled by multiple pulse irradiation of femtosecond laser

**DOI:** 10.1038/s41598-022-27229-0

**Published:** 2023-01-09

**Authors:** Ryota Kiya, Tao Tang, Yo Tanaka, Yoichiroh Hosokawa, Yaxiaer Yalikun

**Affiliations:** 1 Division of Materials Science, Nara Institute of Science and Technology, 8916-5 Takayama-cho, Ikoma, Nara 630-0192 Japan; 2 Center for Biosystems Dynamics Research (BDR), RIKEN, 1-3 Yamadaoka, Suita, Osaka 565-0871 Japan

**Keywords:** Optical manipulation and tweezers, Biotechnology

## Abstract

Femtosecond-laser-assisted cell manipulation, as one of the high throughput cell sorting techniques, is tailored for single-step multiple sorting based on controllable impulsive force. In this paper, femtosecond laser pulses are focused within a pocket structure and they induce an impulse force acting on the flowing objects. The impulsive force is shown to be controllable by a new method to adjust the femtosecond pulse properties. This allows precise streamline manipulation of objects having various physical qualities (e.g., weight and volume). The pulse energy, pulse number, and pulse interval of the femtosecond laser are altered to determine the impulsive force strength. The method is validated in single cell or bead triple-sorting experiments and its capability to perform streamline manipulation in as little as 10 μs is shown. The shift profiles of the beads acting under the impulsive force are studied in order to better understand the sorting mechanism. Additionally, beads and cells with different fluorescence intensities are successfully detected and directed into different microchannels, with maximum success rates of 90% and 64.5%, respectively. To sum up, all results suggest that this method has the potential to sort arbitrary subpopulations by altering the number of femtosecond pulses and that it takes the first step toward developing a single-step multi-selective system.

## Introduction

Sorting particles and cells from heterogeneous suspensions is an essential step in the processing and purification of complex mixtures for subsequent analysis and diagnosis^1–3^. A sorting process is widely used as the first step for many therapeutic and diagnostic practices including drug screening^4^, stem cell investigation^5^, tissue and organ regeneration^6^, and cancer diagnostics and therapy^7–9^. In order to isolate key cells one by one, precise sorting methods employ an external force (acoustic, electric, optical, or jet force) to manipulate the streamline of cells with certain biomarkers.

To date, there are numerous precise sorting methods and applications^3^ that are based on acoustophoresis^10,11^, dielectrophoresis^12,13^, micro-electro-mechanical system (MEMS)^14,15^ and optical methods^16,17^. For example, a traveling surface acoustic wave (TSAW)^18^ has been shown to realize volume-based cell sorting. However, standard acoustic microfluidic devices have the demerit of a high voltage requirement (e.g., > 200 Vpp) and they generate excess heat^19,20^. Due to poor energy conversion, a large portion of the electrical energy used in acoustofluidic sorters is lost as heat which weakens the biocompatibility of acoustofluidic sorters. An alternative is to use dielectrophoresis (DEP) methods that realize cell sorting based on intrinsic dielectric properties of the cells. A non-uniform electric field is employed to displace flowing objects into different balance positions^21^. The side effects^22^ of DEP methods on biophysical properties and viability of cells limit their direct application to cell sorting, and DEP-based sorting methods typically use droplet techniques^23,24^. Additionally, magnetophoresis (MAP) has been shown to realize a high-throughput cell sorting (83 kHz^25^), but labeling cells with magnetic beads or removing labels are laborious and difficult tasks.

The femtosecond-laser-assisted method^26^ offers several advantages over other cell manipulation techniques; it offers high throughput, up to 100 kHz, which far exceeds other approaches, such as DEP (90 Hz^27^), TSAW (4 kHz^10^), and piezoelectric transducer (PZT) (23 kHz^28^) methods. Additionally, it was reported that femtosecond-laser-assisted binary sorting reached a 100% success rate at a high throughput (100 kHz) for polystyrene beads and a 63% success rate at an 83.3 kHz throughput for cells^26^.

In this work, a jet flow is generated by the femtosecond pulse irradiation and the flow acts as an impulsive force on objects, i.e., the femtosecond pulse irradiation generates shock and stress pressure waves, resulting in the jet flow that acts on objects, sorting them. Recent research has demonstrated that this sorting process does not affect cell viability since no heat is generated during it^29^. No physical heating makes it more biocompatible than other methods that use acoustofluidic^19,20^ or thermal-based^30^ microdevice sorting techniques. The impulse force is mainly generated by the multi-photon absorption of water molecules^29^, and no obvious heat accumulation effect is observed^31,32^. Previous research reported that the high repetition rate (> 200 kHz) of ultrashort laser pulses causes limited heat accumulation effects^33^; however, in the present work the repetition rate of the femtosecond laser is 100 kHz which is not enough to cause detectable heat accumulation effects. As a final advantage, the strength of the impulsive force is determined by the pulse number, pulse energy, and pulse intervals of the femtosecond-laser system^29^ which allows for customized streamlining manipulation in a high-throughput system.

In this paper, we present our new method that has the capacity to efficiently manipulate the streamline of individual cells or beads with varying fluorescence intensities into different channel outlets. We implement the method into a femtosecond-laser-assisted triple-selective system. A pocket structure is put on the channel wall next to the fluorescence detection region to ensure that all impulsive forces travel unidirectionally and focus at the sorting region^34^ and to prevent the potential negative influence induced by the laser irradiation on the target. The pocket structure for laser irradiation is integrated into the microchannel to increase the distance (it should be > 10 μm^35^) between the focus point and the target. In addition, the strength and duration of the impulsive force are evaluated as a function of the number of femtosecond pulses by analyzing the streamline profile of the polystyrene beads. Experiments are performed on polystyrene beads and floating cells of different sizes and fluorescence intensities. The results confirm the feasibility of using the femtosecond laser system for high-throughput triple-selective sorting and this work is considered to be a first step toward constructing a high-throughput multi-selective system.

## Results

### Outline of the laser-assisted triple-selective system

**Figure 1:**
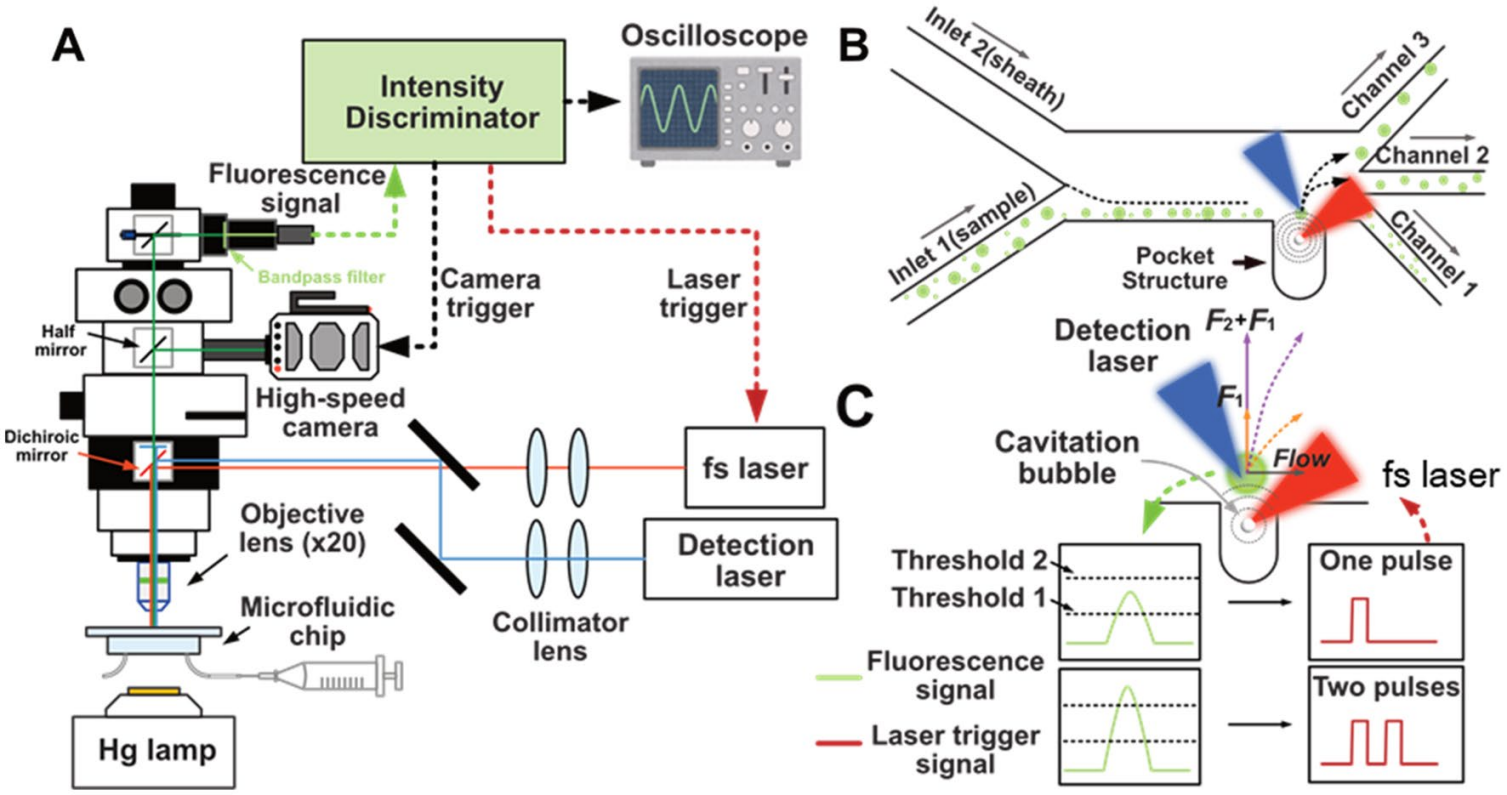
Schematic of the femtosecond-laser-assisted triple-selective system. Micro-objects are subjected to the impulsive force generated by the femtosecond laser (fs laser) and sorted to distinct channel outlets. The impulsive force is determined by the number of femtosecond pulses. (**A**) Fluorescence detection and cell sorting experimental setup. (**B**) Operating concept of the femtosecond-laser-assisted triple-selective system. (**C**) Schematic of the number control of the femtosecond pulses based on different fluorescence intensities.

The schematic diagram of the femtosecond-laser-assisted multi-selective system shown in Fig. 1A shows four primary components: the microfluidic channel, the optical detection system, the intensity discriminator, and the femtosecond-laser system. The detailed parameters of the microchannel are shown in Supplementary Fig. S1. The sample suspension is injected into the microchannel at a flow rate of 10 μL/min, and a sheath flow (130 μL/min) is used to focus the sample flow adjacent to the channel wall (Fig. 1B). When a fluorescent object is detected by the detection laser, a femtosecond pulse is focused in the pocket structure. A rapid pressure drop occurs at the laser focal point and it results in formation of a cavitation bubble (i.e., a vapor bubble). As a result of the expansion of the cavitation bubble, a jet flow is generated and directed onto nearby objects, acting as an impulsive force.

As illustrated in Fig. 1C, a single femtosecond pulse is triggered for objects with a low fluorescence intensity (i.e., > Threshold 1). The impulsive force directs the target object towards the streamline corresponding to the middle channel (Channel 2). If the fluorescence intensity of the cells or beads exceeds a predetermined threshold (i.e., > Threshold 2), double femtosecond pulses are irradiated into the pocket, producing a larger impulsive force and directing the target into Channel 3 (the outermost channel). Additionally, if the fluorescence intensity of objects is too low, no femtosecond pulse is triggered, and all objects pass through Channel 1 (the innermost one) along the original streamline. Based on the fluorescence signals, we used a laboratory-built intensity discriminator in this work to estimate the number of femtosecond pulses. From fluorescence detection to laser triggering, the sorting of single cells and beads takes approximately 10 μs.

### Intensity discriminator

**Figure 2:**
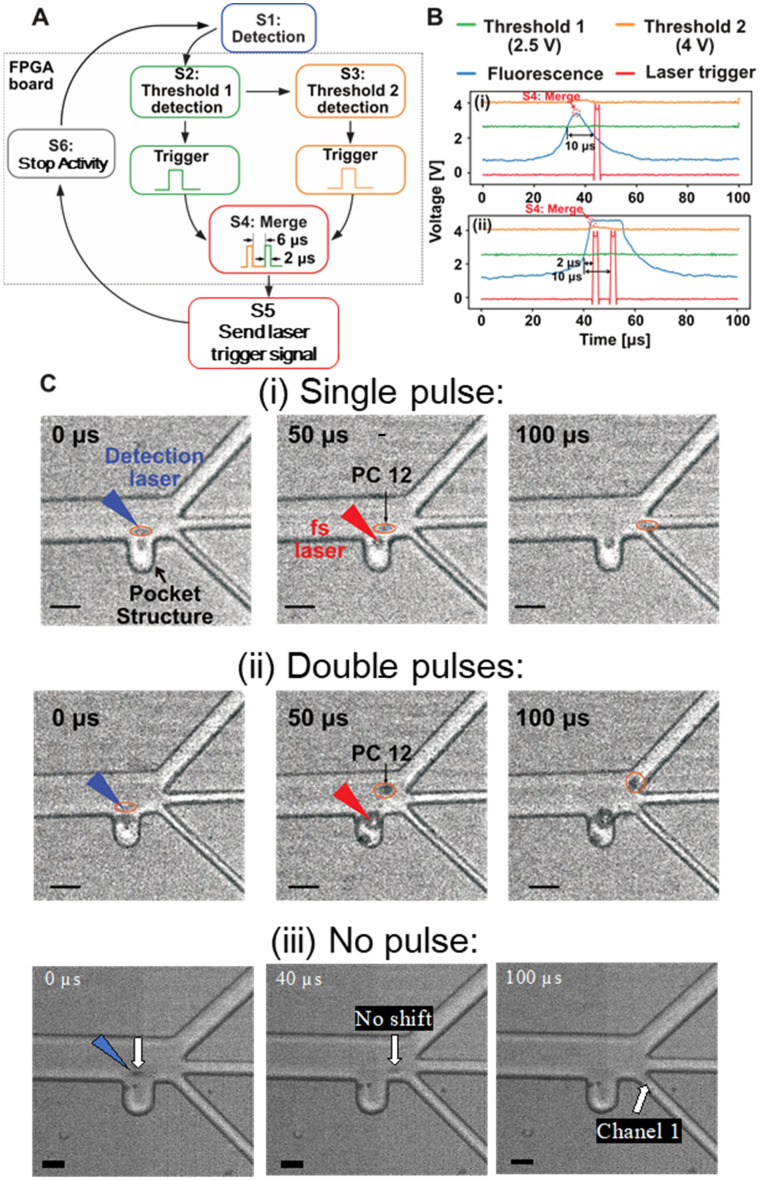
Demonstrations of cell sorting by pulse control. (**A**) Schematic of the intensity discrimination. (**B**) Oscilloscope illustrations showing the fluorescence signal and laser trigger signal in the case of (i) a single pulse and (ii) double pulses. (**C**) Experimental results for PC-12 cells in the case of (i) a single pulse, (ii) double pulses, and (iii) no pulse. The scale bars represent 40 μm.

We used a field-programmable gate array (FPGA) board (Zynq UltraScale + MPSoc XCZU9EG) to implement the intensity discriminator, with lab-developed scripts controlling its operation. The intensity discriminator (Fig. 2A) has six states that are used to calculate the number of femtosecond pulses based on the fluorescence intensity. When the system is in a particular state, a particular action can be performed. The initial state of the detection system is "S1: Detection", meaning that the system is ready to detect a fluorescent object. Once the fluorescent intensity exceeds Threshold 1 (2.5 V), State S2 is initiated, generating a trigger for laser with a width of 2 μs. State S3 is also generated if the maximum fluorescence intensity exceeds Threshold 2 (4 V). In State S4, triggering signals of States S2 and S3 are arranged in order and set with 10 μs and 2 μs delays, respectively. Their merged laser trigger signal is sent from the discrimination system to the femtosecond-laser system at the end of the rising edge (Fig. 2B) of the fluorescence signal, in State S5. After laser irradiation, the system switches to "S6: Stop Activity" to clear all activities and wait for the next fluorescence object to arrive.

We determined two threshold values of 2.5 V and 4 V (Fig. 2B) and demonstrated the sorting steps of the triple-selective system using PC-12 cells (a cell line derived from a pheochromocytoma of the rat adrenal medulla, RCB0009, RIKEN Cell Bank; Tsukuba, Japan) as an example. (The reasons for selecting this as the target are discussed in “Cell sorter analysis”.) To begin, the fluorescence detector transforms the fluorescence intensity into voltage signals using a photomultiplier tube (PMT). In response to a fluorescence voltage greater than 2.5 V, a single femtosecond pulse is focused, and PC-12 cells are directed into Channel 2 (Fig. 2Ci). On the other hand, the PC-12cells are deflected to Channel 3 by double femtosecond pulses when the fluorescence voltage exceeds 4 V (Fig. 2Cii). There is also a waste channel (Channel 1) on the microfluidic chip for storage of cells or beads with little to no fluorescence (Fig. 2Ciii). Because the lifetime of the bubbles is extremely short (μs order), the risk for any kind of flow disturbance caused by these bubbles is low.

### Number of femtosecond pulses

Previous work that we carried out established a binary separation system using a femtosecond laser, in which the laser was focused in the center of the microchannel, adjacent to the center-focused sample stream^36^. We demonstrated that the femtosecond-laser-based cell sorter was highly efficient (i.e., 100 kHz sorting throughput), but there was a risk from direct laser irradiation on bio-samples, which could instantly kill the target cells at such a high pulse energy (≥ 1.0 μJ/pulse). In order to resolve this issue, we switched the laser focal point from the channel center to the pocket structure on the channel wall. In the pocket, the impulsive force induced is less dispersed and can focus on the sorting area^34^.

**Figure 3:**
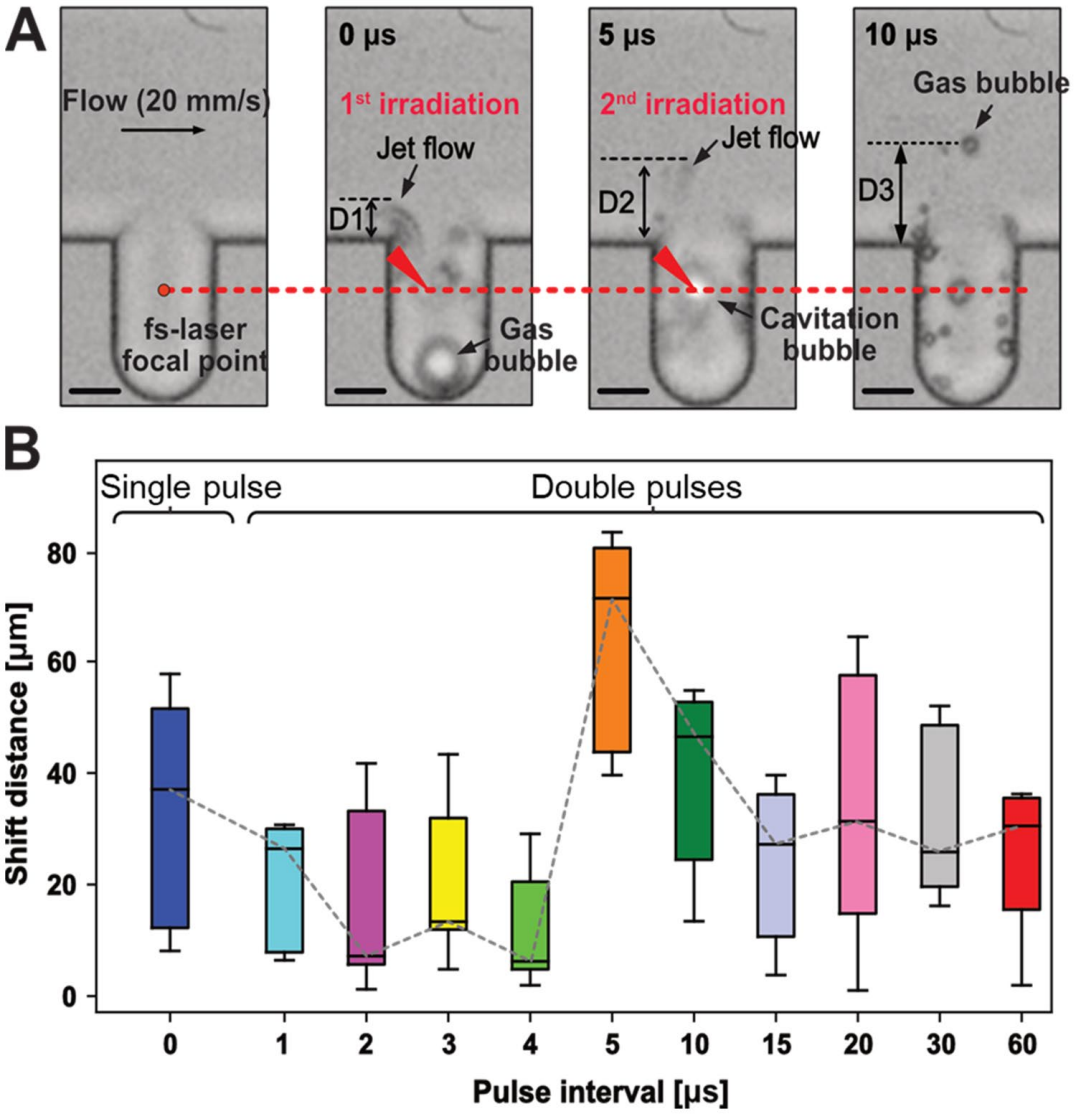
Effects of pulse internal and pulse number on the shift distance. (**A**) Jet flow and a gas bubble generated by femtosecond pulses. The scale bars indicate 20 μm. (**B**) Effects of the pulse interval on the shift distance.

Figure 3A illustrates the temporal evolution of a gas bubble and jet flow around the pocket structure. Smaller gas bubbles are formed if a cavitation bubble collapses and releases dissolved air (gas) into the medium^29^. The cavitation bubble and smaller gas bubbles can also be seen in Fig. 3A. As it is difficult to observe the impulsive force directly, we estimated it by using the position of the gas bubble and jet flow. According to the images presented in Fig. 3A, the jet flow and gas bubble were pushed farther away (i.e., shift distance D2 > 2 × shift distance D1) by the second irradiation pulse than the maximum shift distance (D1) of the jet flow induced by the first irradiation pulse. We hypothesized that two femtosecond laser pulses would produce an impulsive force more than twice as strong as a single femtosecond laser pulse.

Figure 3B illustrates the effects of the pulse interval on the maximum shift distance of the jet flow or gas bubbles, e.g., the shift distance (D3) in Fig. 3A. Double pulses had a smaller shift distance than single pulses when the interval between two pulses was less than 5 μs. In this case, the first femtosecond pulse generated a cavitation bubble that typically lasted longer than 5 μs, so the second femtosecond pulse might focus in the bubble rather than in the fluid. Apparently, the second femtosecond pulse accelerated the collapse of the cavitation bubble and suppressed the jet flow and impulsive force generated by expansion of the cavitation bubble. Additionally, as the impulsive force included both positive and negative forces in the generation and collapse of cavitation bubbles, the jet flow was cancelled out in the superposition of the first and second impulsive forces when the interval was greater than 10 μs^29^. The preferred pulse interval was therefore 5 μs to 10 μs, which could result in a shift distance twice that of a single pulse. For this work, we set the pulse interval to 6 μs because we wanted stable performance of the FPGA board-based discrimination system.

### Streamline manipulation

**Figure 4:**
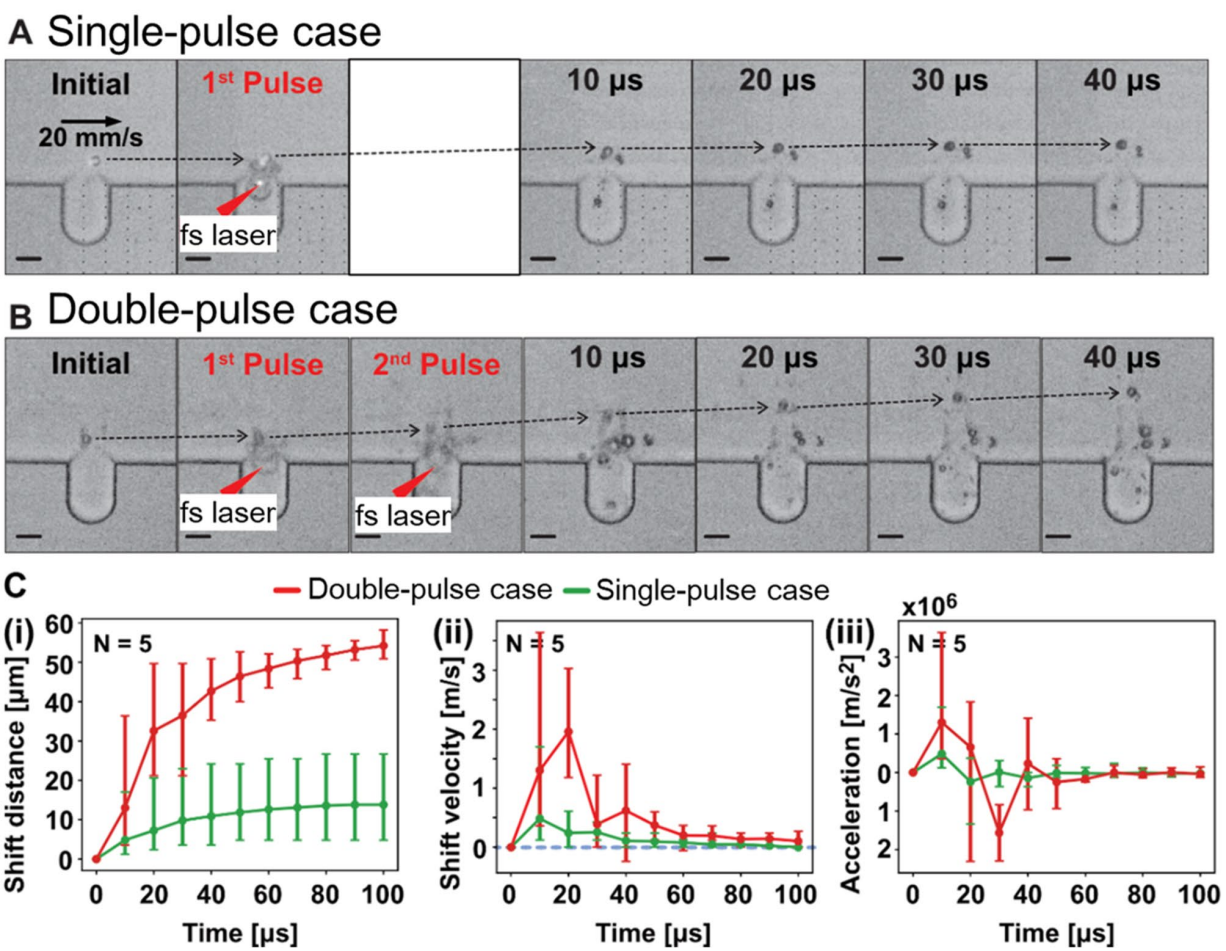
Effects of femtosecond-laser-induced impulsive force on the 10 μm polystyrene beads. (**A,B**) Shifted flow profile of 10 μm beads in response to (**A**) a single femtosecond pulse and (**B**) double femtosecond pulses. (**C**) Time series of (i) shift distance, (ii) velocity, and (iii) acceleration of the beads in single- or double-pulse cases within 100 μs. The scale bars represent 20 μm. The error bar is the standard deviation calculated from five sets of values.

The shift profile of 10 μm polystyrene beads was visualized using a high-speed camera (100,000 fps) in order to assess the effect of impulsive force produced by femtosecond pulses on the streamline. Figure 4 illustrates the shift profile of 10 μm beads when stimulated with single or double femtosecond pulses. For a single pulse, the shifted flow profile (Fig. 4A) had a parabolic shape, with most of the lateral movement occurring over the first 10 μm at approximately 0.53 m/s. For double-pulse excitation, the duration of the impulsive force was significantly longer than that of single-pulse excitation. The beads moved continuously for 40 μs with the double-pulse excitation (Fig. 4B), whereas with a single pulse, the movement was primarily over within 20 μs. Furthermore, two femtosecond pulses generated significantly greater impulsive force than a single femtosecond pulse, which was demonstrated by the greater shift distance of beads at 10 μs for the double-pulse case versus the single-pulse case (Fig. 4A,B). For double-pulse irradiation, impulsive force accumulated, causing jet flow amplification and impulsive force amplification.

Figure 4C shows the shift distance, velocity, and acceleration of five beads over a 100 μs period. Double pulses produced a shift distance that was nearly five times greater than the single pulse (Fig. 4Ci). Hence, the force generated by the double pulses should be higher than the force generated by a single pulse. In Fig. 4Cii, the average moving speed induced by double pulses after 10 s was nearly three times higher than that of the single pulse. The average velocity was still positive for 100 μs in the double-pulse case, whereas it was zero after 40 μs in the single-pulse case. Also, the duration of the impulsive force could be estimated from the acceleration of the beads (Fig. 4Ciii). The impulsive force lasted less than 10 μs before the acceleration returned to zero for the single-pulse case. In comparison, the impulsive force induced by the double pulses did not dissipate after 10 μs, since the acceleration of all five beads remained positive at that time.

### Bead sorter analysis

The performance of the femtosecond-laser-aided triple-selective system was evaluated using three different polystyrene beads (i.e., 3 μm fluorescent beads, 10 μm fluorescent beads, and 10 μm nonfluorescent beads). They were selected to mimic objects of varying volumes and fluorescence intensities. Sample suspension was loaded into the microchannel and focused next to the channel wall, and single beads passed the sorting area at a velocity of 200 mm/s. The energy of the femtosecond pulse was set to 8 μJ/pulse based on our prior experience. The sorting procedure is shown in Supplementary Movies S1 and S2.

**Figure 5:**
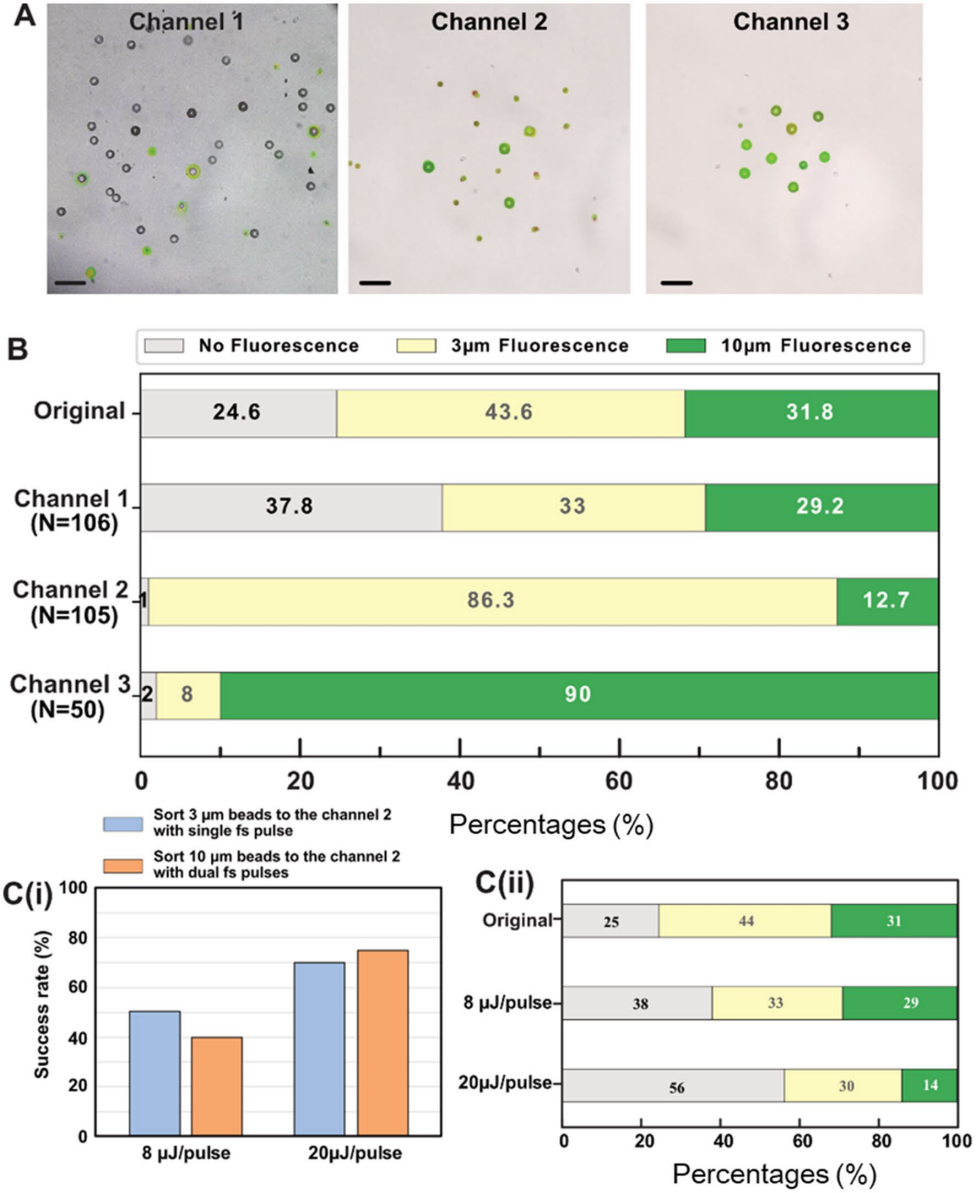
Summarized evaluation of the performance of the femtosecond-laser-assisted triple-selective system using polystyrene beads. (**A**) Photographs of the beads exiting three channels following sorting. (**B**) Percentages of nonfluorescent beads, 3 μm fluorescent beads and 10 μm fluorescent beads to the total number of beads at the outlet of each channel in the original suspension and following sorting. (**C**) (i) Success rate at different pulse energies (n = 20). (ii) Percentages at different pulse energies. The scale bars represent 40 μm.

Figure 5 displays typical sorting results. The liquid from all three channel outlets was used to count the number of beads as each channel output. Each of the images in Fig. 5A was obtained by overlapping eight images in order to increase the number of beads per image for better visualization. Compared to fluorescent beads, nonfluorescent ones were more likely to enter Channel 1 (Fig. 5A) as they could not activate the femtosecond pulses. The percentage of nonfluorescent beads in Channel 1 was increased to 37.8%, compared to 24.6% in the original mixture (Fig. 5B). The output of Channels 2 and 3 showed significant sample enrichment for 3 and 10 μm fluorescent beads, respectively. Since the fluorescent beads differed in size and fluorescence intensity, the voltage signal detected by the fluorescence detector varied. Here, 3 μm fluorescent beads triggered voltage signals between 2.4 and 4 V, while 10 μm fluorescent beads triggered voltage signals greater than 4 V. Using threshold voltages of 2.5 V and 4 V, 3 μm fluorescent beads could induce only one femtosecond pulse, with the beads being directed into Channel 2 with a shift distance of about 10 μm. For 3 μm beads, the initial percentage of 43.6% was enriched to 86.3% in the Channel 2 output. In the case of the fluorescent beads with a 10 μm diameter, a strong fluorescence intensity resulted in double femtosecond pulse triggers, causing greater impulsive forces. Consequently, the shift distance was about 50 μm, and most of the 10 μm beads entered Channel 3. In the Channel 3 output, 10 μm fluorescent beads accounted for 90%, which was significantly higher than the 31.8% in the original mixture.

Comparing the original sample with the percentage of the strongly fluorescent beads in the sample output from Channel 1, we saw that the percentage hardly changed. This indicated that the success rate of this system was low. Therefore we investigated the extraction efficiency at a higher pulse energy condition (the original condition of 8 μJ/pulse was raised to 20 μJ/pulse). From the graph of Fig. 5Ci, we saw that a larger pulse energy improved the success rate. In Fig. 5Cii the extraction efficiency rose from 37.8%, 33% and 29.2% to 56%, 30% and 14%, for nonfluorescent beads, 3 μm fluorescence beads and 10 μm fluorescence beads, respectively. By further increasing the pulse energy, these is a potential for further increasing the extraction efficiency (20 μJ/pulse is the maximum pulse energy possible for our system).

### Cell sorter analysis

We further evaluated the applicability of the developed laser-assisted selective sorting system for the separation of bio-samples by using fluorescently stained and unstained PC-12 cells. We chose PC-12 cells (RCB0009, RIKEN Cell Bank; Tsukuba, Japan) for their ease of culturing, accessing and modifying (they easily differentiate into other cells^37^). Also, PC-12 cells have a wide range of sizes from 3 to 12 μm which allows them to be used for modeling most mammalian floating cells. Due to the different properties (e.g., density) and morphologies (e.g., shape, size and volume) of different types of cells, slight modification of laser conditions is required.

**Figure 6:**
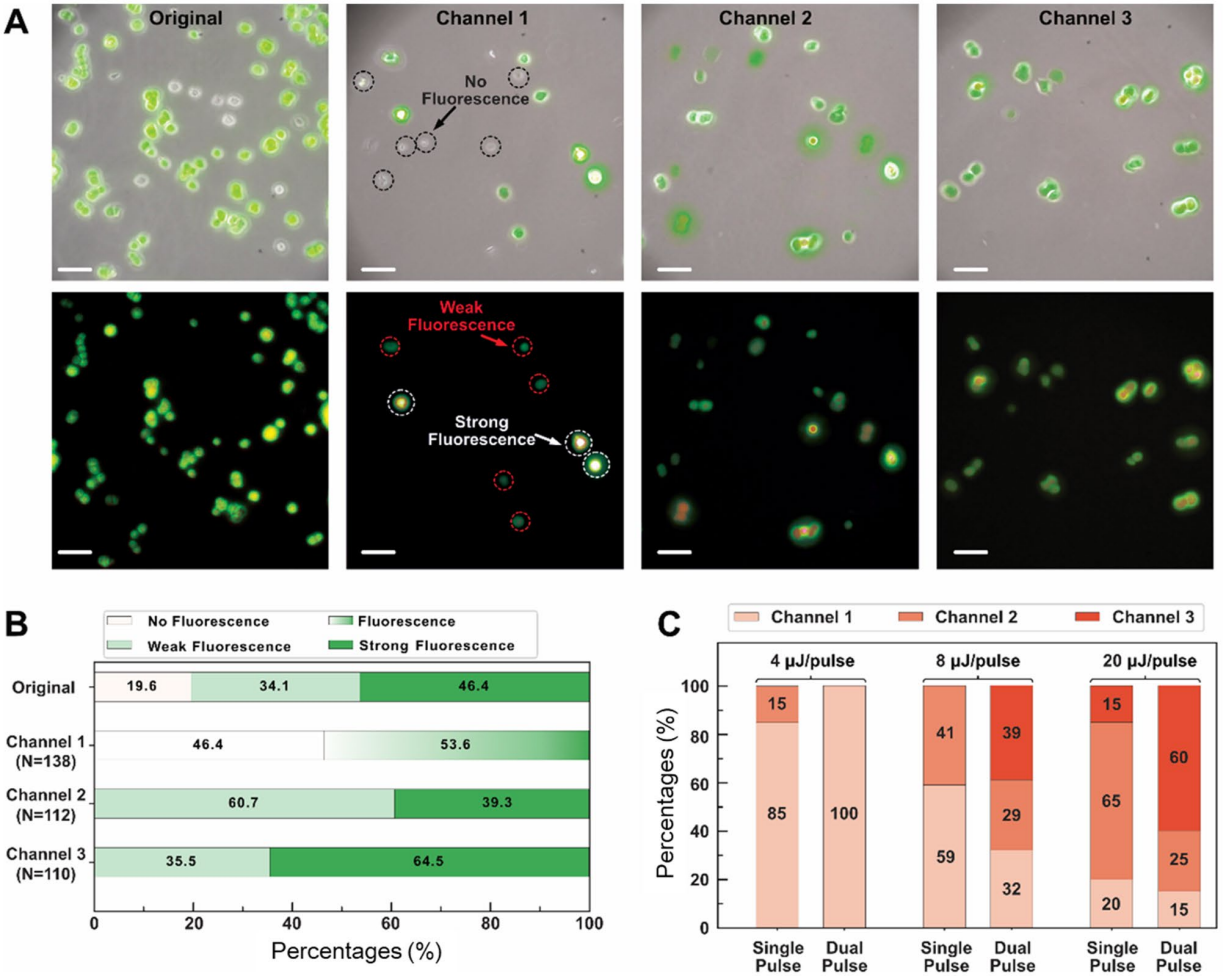
Demonstration for sorting PC-12 cells with varying fluorescence intensities. (**A**) Images of the original mix and the sample as the outputs of Channels 1 to 3. (**B**) Pre- and post-sorting changes in the percentages of nonfluorescent cells, weakly fluorescent cells, and strongly fluorescent cells. The pulse energy for the sorting was 8 μJ/pulse. (**C**) Effect of femtosecond pulse energy on the lateral displacement of PC-12 cells (n = 20 for each condition). The scale bars represent 40 μm.

Bio-samples retain their viability when manipulated by a laser, as evidenced by previous studies^38,39^. Because of the different cell states and sizes among individual PC-12 cells, the fluorescence intensity of each cell should also be different, as shown in Fig. 6A. Smaller cells, for example, are more likely to emit weak fluorescent signals, just as small beads (3 μm) emit less light than larger beads (10 μm). Images in Fig. 6A represent the overlap between fluorescent and nonfluorescent charge-coupled device (CCD) images. We used them to determine the fluorescence intensity and to count cells at the outlet of each channel. In the sorting area, PC-12 cells flowed at a velocity of 200 mm/s during the sorting process. For each of the femtosecond pulses, 8 μJ/pulse was set, as in bead sorting. The sorting procedure is shown in Supplementary Movies S3 and S4.

Cell sorting performance was assessed by measuring the percent ratio of nonfluorescent, weakly fluorescent, and strongly fluorescent cells to the total number of cells in each channel output, as illustrated in Fig. 6B. Because nonfluorescent cells were unable to activate the detection system, they would continuously flow into Channel 1, resulting in 46.4% nonfluorescent cells being found at that channel outlet (19.6% in the original mixture). In the case of weakly fluorescent PC-12 cells (i.e., 34.1% in the original mixture), they were capable of triggering a voltage signal greater than 2.5 V, so a single femtosecond pulse was activated to direct cells into Channel 2. Single-pulse sorting results showed that 60.7% of cells at Channel 2 exit emitted weak fluorescence. Besides, the strongly fluorescent cases were subjected to stronger impulsive forces generated by double femtosecond pulses, and theoretically most of them would be directed into Channel 3. Among PC-12 cells in Channel 3, 64.5% showed strong fluorescence, and 35.5% displayed weak fluorescence. The weakly fluorescent samples in Channel 3 were a result of double femtosecond pulses that were being mistakenly triggered by weakly fluorescent samples.

Figure 6C shows the relationship between the pulse energy of the femtosecond pulse and the sorting performance of the on-chip sorter. With the femtosecond pulse being 8 μJ/pulse, the impulsive force generated by a single femtosecond pulse was insufficient to push the cells into Channel 3. The reason for the sample being directed into Channel 3 should be the effect of double femtosecond pulses, which agrees with our hypothesis explaining the origin of cells with low fluorescence in Channel 3. The intensity of the fluorescence was not uniformly distributed within the cells as was the case with fluorescent beads. As a result, weakly fluorescent cells can trigger double femtosecond pulses when specific areas of the cells are highly concentrated in fluorescent components.

The femtosecond pulse at 8 μJ/pulse was insufficient to shift the streamline of cells to Channel 3 even with a double-pulse stimulus (Fig. 6C). Using double femtosecond pulses, we successfully sorted 39% of the PC-12 cells into Channel 3, which was significantly lower than the success rate for the bead sorter experiment. With regard to sorting the beads, their volume and weight remained constant, and the streamline was predictable when subjected to the impulsive force generated by the femtosecond laser. However, even with the same force, the lateral movement of individual cells is unpredictable and depends on a variety of factors, including the state of the cell cycle, viability, and even osmosis. This dependency problem might be solved by increasing the energy of individual femtosecond pulses. For example, femtosecond pulses of 20 μJ/pulse are more likely to direct cells to Channel 3 via double femtosecond pulses (i.e., single pulse, 15% and double pulses, 60%). The effects of large impulsive forces on cell viability, however, should be considered, which will require further investigation in the future. As well, femtosecond pulses of 4 μJ/pulse were examined, but the force emitted was too weak to dislodge cells from the streamline. Overall, femtosecond pulses cause a streamline shift which is dependent on both the pulse energy and the biophysical properties of individual cells (i.e., cell volume, weight, and distribution of fluorescent components).

## Discussion

Femtosecond pulses were used to manipulate streamlined objects with a high throughput (10 μs in this case) and to sort cells or beads based on fluorescence intensities. A deflection of the streamline of the target objects occurs as a result of the impulsive force generated by the femtosecond pulse irradiation. Objects with different fluorescence intensities are directed to different channel outlets by different impulsive forces. The strength of the impulsive force is determined by three factors, namely, pulse number, pulse energy, and pulse interval of the femtosecond laser. The sorting results on single beads showed the applicability of the method with 90% and 86.3% extraction efficiency for strongly and weakly fluorescent beads, respectively. When single fluorescent cells were sorted, the percentages of the strongly and weakly fluorescent cells (64.5% and 60.7% after sorting) were improved significantly as well (46.4% and 34.1% in the original mixture). Inhomogeneity of cell weight and volume are believed to be responsible for the lower sorting performance of single cells.

Previous studies have demonstrated that impulsive force generated by single femtosecond pulses is reliable and stable for phenotyping mechanism properties of cells on a nanonewton scale^29,40,41^. In other words, for a given femtosecond pulse energy, the impulsive force remains constant. The cavitation detonation increases as the energy of the femtosecond pulse increases, which results in a larger impulsive force^42–44^. By increasing the pulse energy from 4 to 20 μJ/pulse, the streamline manipulation success rate increases from 20 to 80%, as shown in Fig. 6C. While sorting cells, however, it is impossible to adjust the femtosecond pulse energy in real time, and it is imperative to set a constant femtosecond pulse energy before performing experiments. Once the femtosecond pulse energy is set, the impulsive force is stable in still water^29^. The impulsive force induced by the femtosecond pulse in this work was isolated from the fluid flow by the pocket structure^34^.

Increased impulsive forces can result in a large displacement of a target object when the number of femtosecond pulses is increased (Fig. 4). In this work, we determined the number of femtosecond pulses by fluorescence intensity to achieve cell triple-sorting of mixtures. It is theoretically possible to send an object to different streamlines depending on how many pulses are applied. In our view, using a sufficient number of femtosecond pulses will provide a system that is multi-selective and can have an arbitrary number of outputs. Toward this end, we are seeking to relate the pulse count to shift distance by establishing a formula in the future.

Additionally, the pulse interval plays a vital role in determining how impulsive forces accumulate (Fig. 3B). For an interval of less than 5 μs between the first and second femtosecond pulses, the second pulse is focused in the cavitation bubble rather than in the fluid. An insufficient time interval between femtosecond pulses could cause the second cavitation bubble and impulse force to fail, since there might not be enough fluid for cavitation^43,45^. Besides, the impulsive force consists of positive and negative forces generated by the expansion and collapse of the cavitation bubble, respectively^29^. In order to realize the superposition of two positive forces, the interval between femtosecond pulses should be greater than 5 μs. Additionally, positive forces induced by the second pulse are cancelled by the negative forces induced by the first femtosecond pulse in cases longer than 10 μs.

As a result of our experiments, we suggest that a pulse energy between 8 and 20 μJ/pulse would be sufficient to deflect objects flowing in a microchannel. It is possible to specify the number of femtosecond pulses according to the number of subpopulations; two femtosecond pulses, for example, could be used when there are three subpopulations. The time interval could be the same as ours, which is 6 μs, where the preferred time interval is between 5 and 10 μs. Additionally, this method also provides insight into the precise manipulation of flow trajectories and may inspire the design of a single-step, multiple sorting system.

The bubble generated after the cavitation bubble bursts will dissolve after hundreds of μs ^46,47^. And due to the extremely high flow velocity (200 mm/s) of our system, the bubbles generated from the cavitation pass into the channel without disturbing^36,48^.

In our investigation about the minimum detectable fluorescence, the difference among the PC-12 cells when using our self-made PMT based system was about 2 V. And we found that it matched the fluorescence difference between 3 and 10 μm fluorescent beads. Furthermore, most mammalian floating cells have a size range from 3  to 10 μm; therefore we chose these two types of beads. Furthermore, our current laser-assisted triple-selective system is capable of detecting smaller fluorescence differences (< 0.5 V) which can be used to identify other cells with a small fluorescence difference^49^.

## Materials and methods

### Sample preparation

To evaluate the performance of our laser-assisted triple-selective system, 10 μm nonfluorescent polystyrene beads, 3 μm and 10 μm fluorescent beads were used (Polysciences, Inc., Warrington, PA). PC-12 cells (RCB0009) were provided by the RIKEN Cell Bank (Tsukuba, Japan) and used for the evaluation, also. The PC-12 cells were maintained in a humidity incubator (5% CO_2_) at 37 °C with Dulbecco's Modified Eagle Medium (DMEM) containing 10% fetal bovine serum (FBS) and 1% penicillin–streptomycin. Prior to the experiment, the cells were stained for 40 min with green CMFDA dye (FUJIFILM, Tokyo, Japan), and cells with a fluorescent label were mixed with unlabeled cells at a ratio of 1:4 to serve as the bio-samples for sorting.

### Microfluidic device

The polydimethylsiloxane (PDMS) device was fabricated using a master mold with negative photoresist (Su-8, 3010; Tokyo Ohka Kogyo, Tokyo, Japan) on a silicon water (4-inch diameter). The PDMS (SYLGARD 184; Dow Corning, Midland, MI) was mixed at a base-to-curing agent ratio of 10:1 w/w. To cure the polymer, we poured the PDMS mixture over the master mold, degassed it, and baked it for 3 h at 80 °C. The PDMS microchannel part was then peeled off the master and cleaned using adhesive tape. Finally, a cleaning apparatus (CY-P2L-B Plasma Cleaner; Zhengzhou CY Scientific Instrument Co., Ltd, Zhengzhou, China) was used for an oxide plasma treatment of the PDMS microchannel part and the glass slide (76 × 26 × 0.8 mm^3^, borosilicate glass) for 1 min at 90 W prior to bonding.

The microfluidic chip device featured two inlets and three outlets. In the sorting region, the microchannel was 20 μm in height and 70 μm in width, and it had a pocket structure (40 μm wide and 60 μm long) on the channel wall. The parameters are presented in detail in Supplementary Fig. S1. The sample fluid was injected through inlet 1 (20 μm wide) at a flow rate of 10 μL/min. To focus cells or beads alongside the microchannel wall, a sheath flow of deionized water was pumped into inlet 2 (40 μm wide). There were three outlet channels, with respective widths of 15 μm, 15 μm, and 40 μm. Regarding the pocket structure, previous studies have shown that it can reduce the risk of direct laser irradiation on bio-sample^50,51^, as well as increase the impulsive force at the sorting area^34^.

### General equipment

A motorized microscope stage (BIOS-L101S OptoSigma, Tokyo) was used to mount the microfluidic chip upside down on an upright microscope (BX53 Olympus, Tokyo). For sample loading (10 μL/min) and sheath flow loading (130 μL/min), two syringe pumps were used (Harvard Apparatus, Massachusetts, Holliston). Additionally, a high-speed camera (Berwyn, Pennsylvania, U.S.Phantom V1211, Ametek, Berwyn, PA, USA) was employed to capture the object motion over the entire period of single cell or bead sorting at a frame rate of 100,000 fps.

For fluorescence detection, an object lens (UPlanFL, Olympus; 20× , 0.5 NA air objective) was used to focus the laser beam onto single cells or beads as they flowed through the sorting area. The laser was a diode-pumped solid-state laser (wavelength 488 nm, 40 mW). The fluorescence intensity was converted into voltage signals using a PMT (Hamamatsu Photonics, Hamamatsu, Japan). We used a bandpass filter with 530 nm central wavelength and a nominal band width of 40 nm in conjunction with a pinhole to reject undesired wavelength bands.

For cell sorting, a regeneratively amplified ytterbium femtosecond pulse laser (Spirit One, Spectra-Physics, Andover, MA, USA) was used. A 500 kHz repetition rate was set for the femtosecond-laser system. This generated femtosecond pulses train with a center wavelength of 1040 nm. Pulse pickers were equipped with acousto-optic modulators, which were controlled by the lab-made intensity discriminator to select a single-shot pulse from the pulse train. The femtosecond pulse was then focused within the pocket structure using the same objective lens used for fluorescence detection^34^.

### Pocket structure

Our past paper reported the function and mechanism of the pocket structure when increasing the impulsive force^34^. Cavitation occurs when the pocket structure provides enough space for a bubble to expand, and the largest impulsive force occurs when the bubble expands to the maximum. Regarding the pocket structure (length, 60 μm; width, 40 μm), we designed a straight pocket in order to provide enough space for cavitation bubbles to form and expand. When the femtosecond pulse was focused within the pocket, the streamline was manipulated by the impulsive force and the jet flow (Supplementary Fig. S3A). Regardless of pocket length, the pocket width of 40 μm was proven to be capable of stabilizing and maximizing the impulsive force (Supplementary Fig. S3B). The pocket length served to suppress the reflection of impulsive forces. Additionally, Supplementary Fig. S3C shows the dependence of the streamline shift on the laser focal point within the pocket structure. There was a significant streamline shift when the laser focal point approached the pocket entry. It should yield a larger impulsive force if the laser is focused closer to the pocket entry. In this case, the laser was focused about 10 μm from the pocket entry.

## Supplementary Information

Supplementary Video 1.

Supplementary Video 2.

Supplementary Video 3.

Supplementary Video 4.

Supplementary Information.

## Data Availability

The datasets used and/or analyzed during the current study are available from the corresponding author on reasonable request.
