# Supplementary material for: Microsecond cell triple-sorting enabled by multiple pulse irradiation of femtosecond laser
Source: Sci Rep. 2023 Jan 9;13:405. doi: 10.1038/s41598-022-27229-0 (PMC9829734; doi:10.1038/s41598-022-27229-0)
Supplement: Supplementary file 5 — Supplementary Information. [file 41598_2022_27229_MOESM5_ESM.docx]

Supplementary Materials for

**Microsecond cell triple-sorting enabled by multiple pulse irradiation of femtosecond laser**

Ryota Kiya^1‡^, Tao Tang^1‡^, Yo Tanaka^2^ Ako Hidekazu^1^, Yoichiroh Hosokawa^1^, and Yaxiaer Yalikun^1,2*^

‡ Both authors contributed equally to this work.

*Corresponding author. Email: [yaxiaer@ms.naist.jp](mailto:yaxiaer@ms.naist.jp)


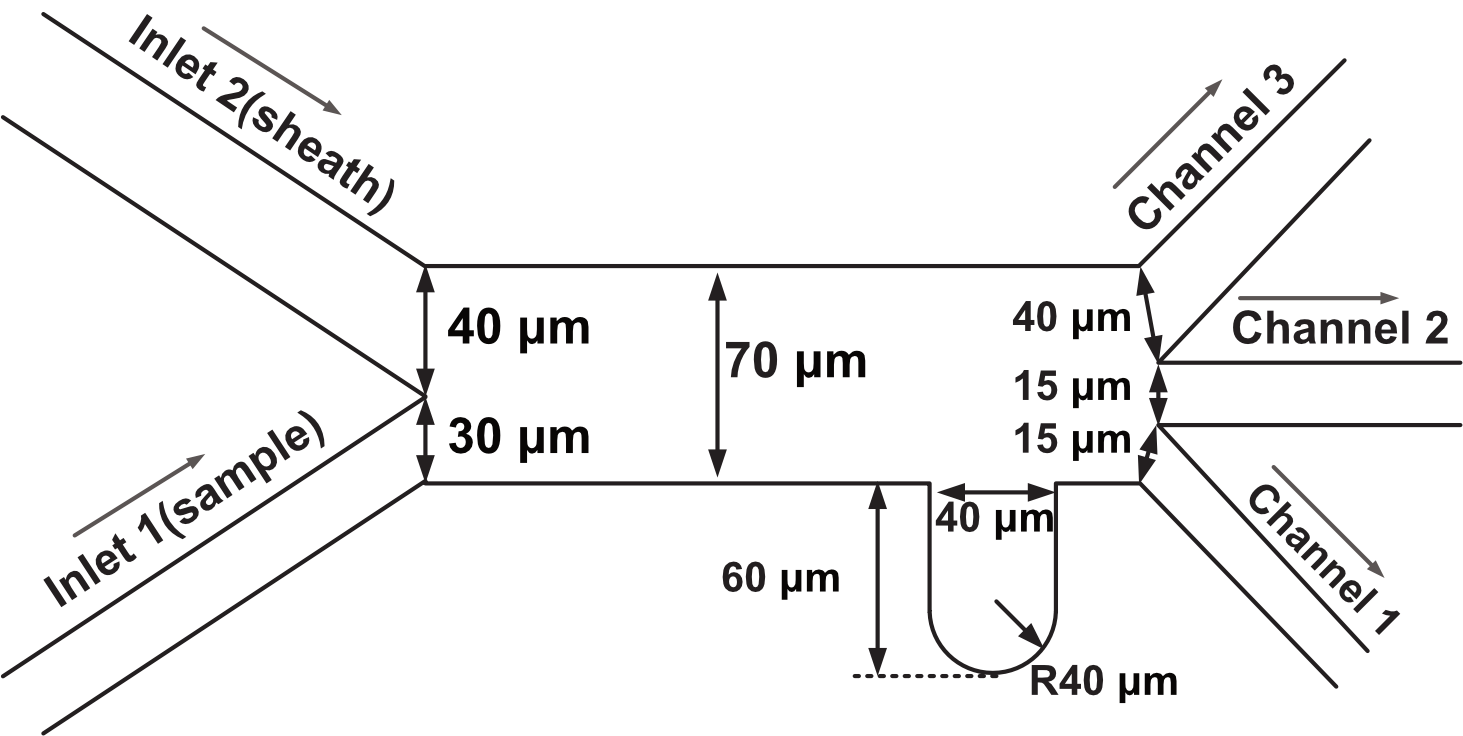
 Fig. S1. Dimensions of the microfluidic channel.

## Impulsive force emission and cavitation bubble

A focused fs pulse causes an ablation plasma to form in fluids within a few hundred picoseconds (*1*), resulting in cavitation bubbles. Cavitation bubbles in fluids are typically characterized by a series of damping activities (*2*, *3*) (e.g., formation, expansion, shrinking, and rebound). In Fig. S2A, while the damping oscillation is occurring, the pressure generated by shock and stress causes the impulsive force (*4*–*6*) and the pressure takes aways the majority of the dynamic energy of the cavitation bubbles (70% ~ 90%) (*7*) converted to the pressure. In addition, the cavitation bubble generates a positive impulsive force when it expands and a negative impulsive force when it shrinks (see Fig. S2B).


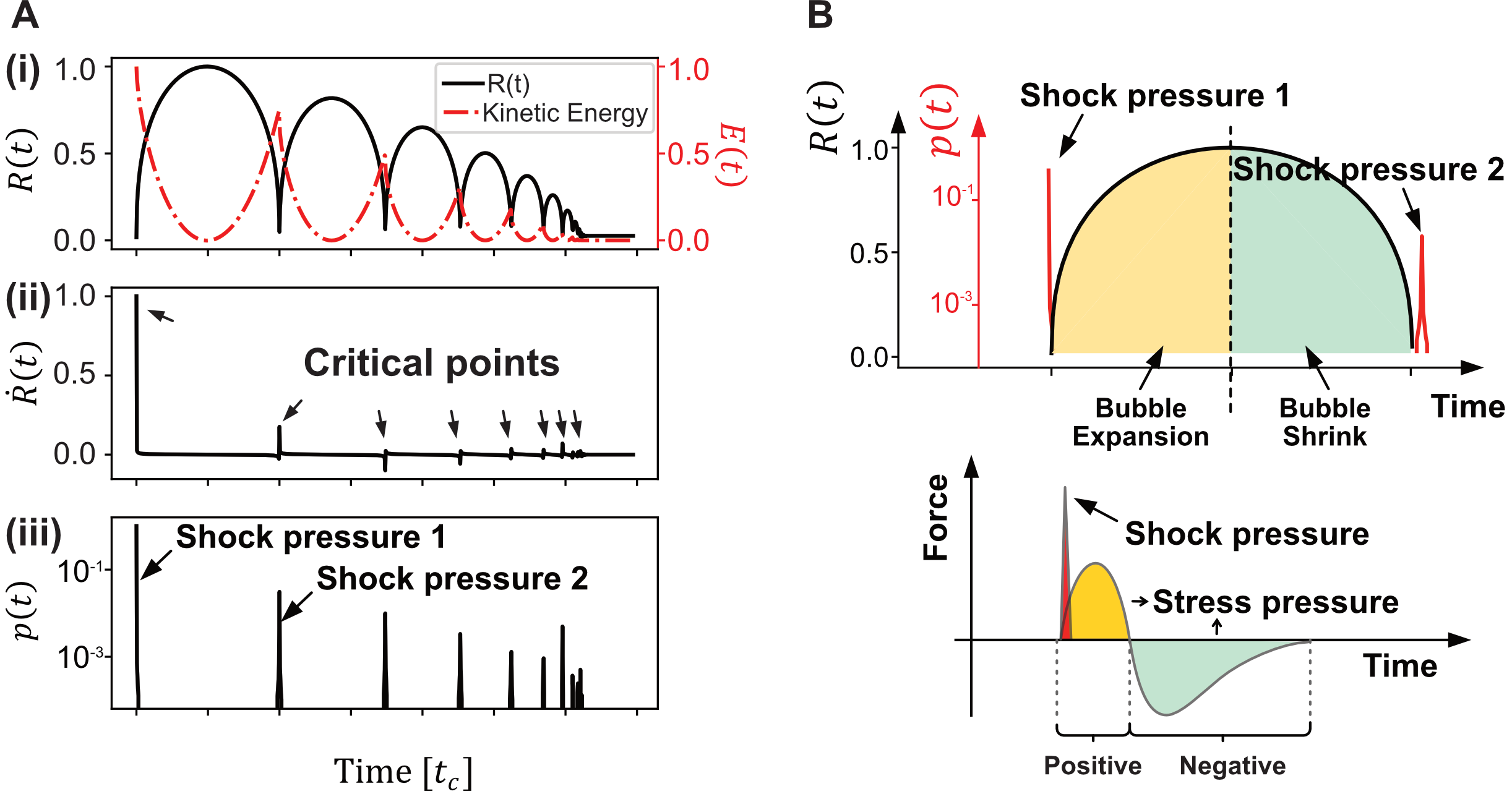


**Fig.S2: Time evolution of the cavitation bubble dynamics.** (A) Numerical simulation of bubble dynamics: (i) Time evolution of bubble radius ($R\left( t \right)$ - solid line) and kinetic energy ($E\left( t \right)$ - red dashed line); (ii) Time evolution of the velocity ($\dot{R}\left( t \right)$) of bubble membrane; (iii) Semi-log graph of the time evolution of the pressure ($p\left( t \right)$) at the bubble wall. (B) Schematic presentation of femtosecond laser-induced shock pressure and stress pressure across a cycle of the bubble oscillation.

The Rayleigh-Plesset equation approximates the oscillations of the cavitation in a compressible liquid as a model (*8*):

$R\ddot{R}+\frac{3}{2}\dot{R}^{2}=p_{g0}\left( \frac{R_{0}}{R} \right)^{3\lambda}-1-\frac{2We}{R}-\frac{4}{Re}\frac{\dot{R}}{R}$ (1)

Where radius $R$ is a function of time ($R\left( t \right)$), and $R_{0},$ $\dot{R}$ and $\ddot{R}$ denote the initial radius, velocity, and acceleration of the bubble, respectively. The initial gas pressure inside the bubble, denoted by $p_{g0}$, is calculated using the hydrostatic pressure ($p_{\infty}$), and the vapor pressure ($p_{v}$) through the following formula: $p_{g0}=\varepsilon\cdot\left( p_{\infty}-p_{v} \right)$. The Weber number ($We$) is defined as $We={\left( p_{\infty}-p_{v} \right)R_{0}}/\gamma$ using gas adiabatic index ($\gamma$).

The instantaneous energy loss can be calculated by differentiating the bubble radius and expressed as (*4*):

$E=\frac{p_{g0}V_{0}}{\kappa-1}\left( \frac{V_{0}}{V} \right)^{\kappa-1}+\gamma A-\left( p_{\infty}-p_{v} \right)V+2\pi\rho R^{3}\dot{R}^{2}$ (2)

Where $A$ and $V$ detnote the surface area and volume of the bubble, respectively. $\kappa$ is the polytropic index of the gas bubble.

Typically, when a cavitation bubble expands (*3*), this results in a positive pressure (i.e., shock pressure) being applied to neighboring liquids, forming the impulsive positive force. On the other hand, the shrinkage of the cavitation bubble leads to the negative impulsive force. On the outside of bubble membrane, the pressure ($p$) over period of the damping oscillation of the bubble is expressed as (*9*):

$P=\frac{3}{2}\dot{R}^{2}\rho-p_{\infty}\left( 1-\frac{R_{0}^{3}}{R^{3}} \right)$ (3)

All parameters (*4*, *10*) in Eqs. (1-3) are determined as $R_{0} = 0.1 mm$, $\varepsilon=100$, $p_{\infty}=100 kPa$, $p_{v}=2.33 kPa$, $\gamma=0.073 N\cdot m^{-1}$, $\rho=1000 kg\cdot m^{-3}$, $\lambda=1.4,$ $\kappa=$ 1.667 and $Re = 50.$ Besides, The radius ($R$) and velocity ($\dot{R}$) of the bubble membrane can be determined by integrating Eq. (1) with the Runge-Kutta method of order 5(4) (*11*). Therefore, we can determine the kinetic energy (Eq. 2) and emission pressure (Eq. 3) of the cavitation bubble system. Because all equations are approximate descriptions of bubble dynamics, timescale and amplitude have no actual physical meaning. The parameters (e.g.,$R\left( t \right), \dot{R}\left( t \right),E\left( t \right) and p\left( t \right)$) in Fig. S2A are therefore normalized into dimensionless parameters based on their maximum value over time. The time scale ($t$) is normalized based on the duration ($t_{c}$) of the first oscillation cycle. Fig. S2A(i) shows the time evolution of the radius and kinetic energy of the cavitation bubble. Kinetic energy is clearly inversely proportional to the radius function and reaches a minimum when the radius reaches a maximum. Each damping oscillation cycle leads to a significant reduction in the overall energy of the bubble (*12*–*15*), and therefore, it is not necessary to consider the impulsive forces generated by the bubble damping oscillation after the first cycle.


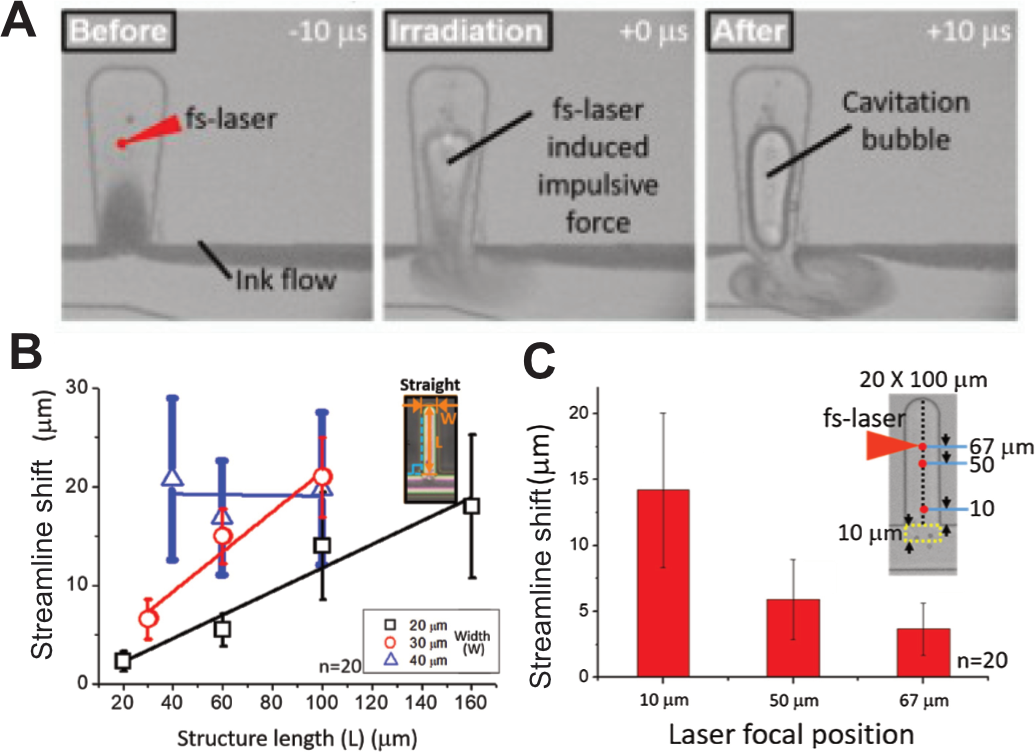


**Fig.S3: Amplification of pocket structure on the impulsive force (fs-laser: femtosecond laser).** (A) Illustration of streamline shift using ink. (B) Effects of the length and width of the pocket on the streamline manipulation. (C) Effects of the location of focal point on the streamline shift. Figures are adopted from Ref. (*16*).

## FPGA code

Git hub link

<https://github.com/oooosya/R.-KIya.git>

Reference:

1. Y. Hanada, K. Sugioka, I. Miyamoto, K. Midorikawa, in *Applied Surface Science* (2005).

2. Q. Wang, W. Liu, A. M. Zhang, Y. Sui, Bubble dynamics in a compressible liquid in contact with a rigid boundary. *Interface Focus*. **5**, 1–12 (2015).

3. H. Nazari-Mahroo, K. Pasandideh, H. A. Navid, R. Sadighi-Bonabi, How important is the liquid bulk viscosity effect on the dynamics of a single cavitation bubble? *Ultrasonics Sonochemistry*. **49**, 47–52 (2018).

4. Q. Wang, Local energy of a bubble system and its loss due to acoustic radiation. *Journal of Fluid Mechanics*. **797**, 201–230 (2016).

5. J. Rapet, Y. Tagawa, C. D. Ohl, Shear-wave generation from cavitation in soft solids. *Applied Physics Letters*. **114** (2019), doi:10.1063/1.5083141.

6. E. A. Brujan, A. Vogel, Stress wave emission and cavitation bubble dynamics by nanosecond optical breakdown in a tissue phantom. *Journal of Fluid Mechanics*. **558**, 281–308 (2006).

7. R. Timm, Optical and acoustic investigations of the dynamics of laser-produced cavitation bubbles near a solid boundary. *Journal of Fluid Mechanics* (1989), doi:10.1017/S0022112089002314.

8. M. S. Plesset, A. Prosperetti, Bubble Dynamics and Cavitation. *Annual Review of Fluid Mechanics*. **9**, 145–185 (1977).

9. M. Farhat, A. Chakravarty, J. E. Field, Luminescence from hydrodynamic cavitation. *Proceedings of the Royal Society A: Mathematical, Physical and Engineering Sciences*. **467**, 591–606 (2011).

10. K. Manmi, Q. Wang, Acoustic microbubble dynamics with viscous effects. *Ultrasonics Sonochemistry*. **36**, 427–436 (2017).

11. J. R. Dormand, P. J. Prince, A family of embedded Runge-Kutta formulae. *Journal of Computational and Applied Mathematics* (1980), doi:10.1016/0771-050X(80)90013-3.

12. G. Huang, M. Zhang, L. Han, X. Ma, B. Huang, Physical investigation of acoustic waves induced by the oscillation and collapse of the single bubble. *Ultrasonics Sonochemistry*. **72**, 105440 (2021).

13. J. P. Caltagirone, S. Vincent, C. Caruyer, A multiphase compressible model for the simulation of multiphase flows. *Computers & Fluids*. **50**, 24–34 (2011).

14. M. Zhang, Q. Chang, X. Ma, G. Wang, B. Huang, Physical investigation of the counterjet dynamics during the bubble rebound. *Ultrasonics Sonochemistry*. **58**, 104706 (2019).

15. X. Ma, X. Zhao, B. Huang, X. Fu, G. Wang, On study of non-spherical bubble collapse near a rigid boundary. *Journal of Hydrodynamics 2019 32:3*. **32**, 523–535 (2019).

16. Z.-Y. Hong, K. Okano, D. di Carlo, Y. Tanaka, Y. Yalikun, Y. Hosokawa, High-speed micro-particle manipulation in a microfluidic chip by directional femtosecond laser impulse. *Sensors and Actuators A: Physical*. **297** (2019), doi:10.1016/j.sna.2019.111566.

Movie S1. Bead sorting into channel 2 with single fs pulse

Movie S2. Bead sorting into channel 3 with double fs pulses

Movie S3. Cell sorting into channel 2 with single fs pulse

Movie S4. Cell sorting into channel 3 with double fs pulses
